# NO DIFFERENCE IN SUBJECTIVE SLEEP MEASUREMENTS BETWEEN MIDDLE-AGED AND OLDER ADULTS

**DOI:** 10.1093/geroni/igad104.2923

**Published:** 2023-12-21

**Authors:** Sally Paulson, Michelle Gray, Megan Jones, Anthony Campitelli, Cody Diehl, Kelsey Bryk, Jordan Glenn, Joshua Gills

**Affiliations:** St. Elizabeth Healthcare, Edgewood, Kentucky, United States; University of Arkansas, Fayetteville, Arkansas, United States; University of Arkansas, Fayetteville, Arkansas, United States; University of Arkansas, Fayetteville, Arkansas, United States; University of Arkansas, Fayetteville, Arkansas, United States; Neurotrack, Redwood City, California, United States; Neurotrack, Redwood City, California, United States; Rutgers University, Newark, New Jersey, United States

## Abstract

With age, sleep quality tends to decline. Poor sleep quality is associated with adverse physical and mental health outcomes, especially in the aging population. The purpose was to compare subjective sleep quality measurements between middle age (45-64 years) and older adults (over 65 years). Participants (N=200) completed the Pittsburgh Sleep Quality Index (PSQI) and were classified by age as either middle age (MID: n=105, 71.4% female, M±SD: age: 55.5±5.8 years; mass: 85.3±17.4 kg, height: 168.4±9.2 cm, education: 18.2±3.6 years) or older adults (OLD: n=95, 76.8% female, M±SD: age: 69.0±2.9 years; mass: 80.6±14.9 kg, height: 166.2±8.9 cm, education: 17.9±3.5 years). The PSQI Component and Global Scores were calculated. Data were analyzed using descriptive statistics, crosstabulations, and independent samples t-tests (p ≤ .05). The results showed there was no difference (p < .05) between the two age groups on subjective sleep measures, including sleep quality, latency, duration, and disruptions. The average Global score for the MID and OLD adults was 6.3±3.2 and 6.0±3.6, respectively. Only 17% of MID adults and 25% of OLD adults were classified as good quality sleepers (PSQI Global score ≤ 4) and 21.9% and 30.5% rated their sleep quality as very good, respectively. While previous research has shown deterioration in subjective sleep quality measures occurs with age, there was not a difference in subjective measures between middle age and older adults in the present investigation. Further analysis is warranted to examine additional factors impacting sleep quality such as physical activity levels, co-morbidities, and living situation.

